# High Prevalence of Metabolic Syndrome and Its Related Demographic Factors in North of Iran: Results from the PERSIAN Guilan Cohort Study

**DOI:** 10.1155/2021/8862456

**Published:** 2021-03-29

**Authors:** Mohammadreza Naghipour, Farahnaz Joukar, Hossein-Ali Nikbakht, Soheil Hassanipour, Mehrnaz Asgharnezhad, Morteza Arab-Zozani, Fariborz Mansour-Ghanaei

**Affiliations:** ^1^Gastrointestinal and Liver Diseases Research Center, Guilan University of Medical Sciences, Rasht, Iran; ^2^GI Cancer Screening and Prevention Research Center, Guilan University of Medical Sciences, Rasht, Iran; ^3^Social Determinants of Health Research Center, Health Research Institute, Babol University of Medical Sciences, Babol, Iran; ^4^Caspian Digestive Diseases Research Center, Guilan University of Medical Sciences, Rasht, Iran; ^5^Social Determinants of Health Research Center, Birjand University of Medical Sciences, Birjand, Iran

## Abstract

**Introduction:**

The prevalence of metabolic syndrome has increased in recent decades around the world and is currently reaching epidemic levels as it is a major public health and clinical concern. The aim of this study was to evaluate the prevalence of metabolic syndrome and its related demographic factors in a population-based study.

**Methods:**

In this cross-sectional study, the target population consisted of 10520 individuals aged 35–70 years in Phase 1 of the Persian Guilan cohort study (Guilan site/Some'e Sara) that was conducted in 2014–2017. Demographic, anthropometric, blood pressure, and biochemical data were used in this study. The IDF definitions were used to diagnose the metabolic syndrome.

**Results:**

The prevalence of the syndrome according to IDF and ATP definition was 42.87% (95% CI: 41.92–41.81) and 40.68% (95% CI: 39.74–41.62), respectively. The prevalence of components for central obesity, high triglyceride, HDL cholesterol, blood glucose, and hypertension components was 75.8%, 43.1%, 40.6%, 39.2% and 37.9%, respectively. All demographic variables were related to the syndrome, and among them age, gender, and residence were identified as independent and strong predictive variables in the regression model. More than 92% of the population had at least one component of the syndrome.

**Conclusion:**

The results of the study show a high prevalence of metabolic syndrome risk factors. It is essential to educate healthy lifestyle behaviors and further health education in the high-risk groups identified in this study, especially the elderly, women, and rural residents.

## 1. Introduction

Metabolic syndrome (MetS) is defined as a set of risk factors such as central obesity, insulin resistance, dyslipidemia, and hypertension that increase the risk of type 2 diabetes mellitus, cardiovascular disease, cancer, and premature death [1, 2]. It is also an important predictor of diabetes and is a simple tool for predicting subsequent CVD [3–6]. This syndrome imposes heavy costs on the health care system and is associated with reduced quality of life [7]. MetS is associated with conditions such as abnormal waist circumference, high fasting blood glucose, high triglycerides, high systolic and diastolic blood pressure, and low HDL. But, in clinical practice, there are different definitions for MetS. In the WHO's definition, high blood sugar plus two abovementioned factors are considered to be a metabolic syndrome [8]. Following strong evidence to support the role of central obesity as a major contributor to MetS and given the significant ethnic differences in the definition of obesity, the World Diabetes Federation set another set of diagnostic criteria for MetS to highlight the role of central obesity and ethnic differences that included obesity plus having two abovementioned factors [9, 10].

The prevalence of MetS has increased in recent decades around the world and has reached an alarming level as it is a major public health and clinical concern [11]. In West and Asian populations, cardiovascular morbidity and mortality are two to five folds higher in people with this syndrome [12–14]. In a systematic review and meta-analysis study, the prevalence of metabolic syndrome in Iran according to ATPIII and IDF was reported 36.9% and 34.6%, respectively [15].

While metabolic components are likely to overlap [16, 17], abdominal obesity has been an independent predictor of new onset of individual MetS components in the longitudinal study [18]. The rate of metabolic syndrome is increasing in line with the increasing prevalence of obesity in developed countries and will increase by 33% over the next two decades and that 51% of the population will be obese by 2030 [19]. There is a significant association between obesity, central obesity with hypertension, diabetes, hyperlipidemia, and MetS. In addition, various demographic factors are associated with MetS. Xi and colleagues have reported variables such as female gender, aging, overweight or obesity, and urbanization as predictors of metabolic syndrome [20]. In another study in Iran, variables such as female gender, increased age, and low literacy were associated with an increased risk of metabolic syndrome [21]. There are also various factors affecting the metabolic syndrome that the contribution of each needs to be examined to different ethnicities.

Assessment of population health status, evaluation of ongoing health plans, attention to specific lifestyles in each area, and reliable information on metabolic risk factors are needed. Therefore, the aim of this study was to evaluate the prevalence of MetS and its related demographic factors using the information obtained from the first phase of Persian cohort of Guilan province.

## 2. Methods

This is a cross-sectional study, and its data are part of a Persian cohort study of Some'e Sara city in Guilan province which includes 10520 adults between 35 and 70 years. Some'e Sara Cohort is a subset of the National PERSIAN Cohort in Iran [22]. Details of Guilan Cohort Profile Previously published with the details [23]. Some'e Sara city is located at Guilan province, north of Iran. The main ethnicities of this region are Gilak.

The data collection at the cohort center consisted of registration procedures, laboratory sampling, anthropometric characterization, and completion of questionnaires, respectively. Eligible individuals were included in the study after completing the informed consent form. The cohort information used in this study was age, gender, place of residence, marital status, current education, and current employment. Anthropometric indices also included weight (kg), height, and waist circumference (cm). The participants were divided into the following groups in terms of body mass index: low weight (BMI <18.5 kg/m^2^), normal weight (BMI = 18.5–24.99 kg/m^2^), overweight (BMI = 25–29.9 kg/m^2^), and obese (BMI ≥30 kg/m^2^). Blood biochemical parameters such as fasting plasma glucose (FPG), triglyceride (TG), amd high-density lipoprotein cholesterol (HDL-C) were used as components of the MetS. Blood pressure was also recorded on the right and left arm.

The prevalence of metabolic syndrome was based on the International Diabetes Federation (IDF) [9] and the Adult Treatment Panel (ATP) III definitions [24], but the IDF definition was considered as the main criterion for calculating the prevalence of the components of the syndrome and performing analyzes. According to the ATPIII definition, a patient must have at least three of the five cardiovascular risk factors at the same time to be diagnosed for the syndrome. The components of the metabolic syndrome include: (1) waist circumference that differs in the two definitions above. In the ATP definition, this component is ≥102 cm for men and ≥88 cm for women, and in the IDF definition is ≥94 cm for men and ≥80 cm for women; (2) high triglyceride (≥150 mg/dl); (3) lower HDL cholesterol (<40 mg/dl in men and <50 mg/dl in women); (4) high blood pressure (Systolic BP ≥130 mmHg and diastolic BP ≥85 mmHg) or previously diagnosed hypertension; (5) high fasting plasma glucose (≥100 mg/dl or previous diagnosis of type 2 diabetes).

Validation of other measurements such as blood pressure instruments has been performed in previous studies [25–29].

### 2.1. Ethical Consideration

The present study was approved by the Ethics Committee of Guilan University of Medical Sciences (IR.GUMS.REC.1397.156).

### 2.2. Statistical Analysis

Demographic characteristics of patients were provided with descriptive statistics such as mean (standard deviation) and frequency (relative frequency). The prevalence of metabolic syndrome and its components was calculated with 95% confidence interval. Logistic regression was used to analyze the relationship between different risk factors of metabolic syndrome, and Pearson correlation coefficient test was used to investigate the relationship between different parameters. Data were analyzed using Stata version 11 software (Stata Corp., College Station, TX, USA). *P* values less than 0.05 were considered as statistically significant.

## 3. Results

**Figure 1:**
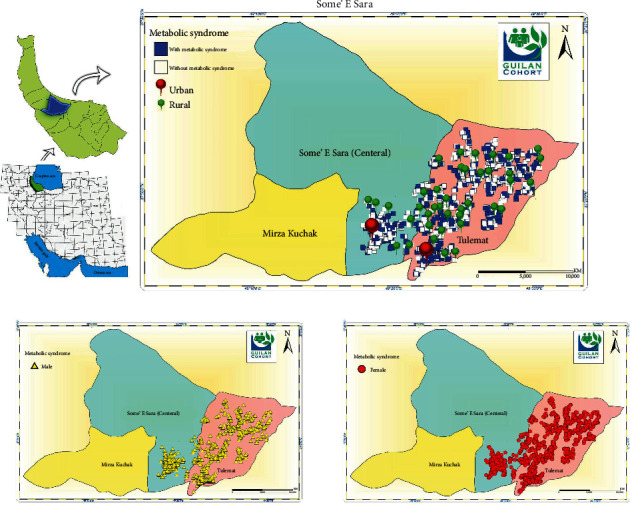
Geographic distribution of metabolic syndrome based on IDF definition in Some'e Sara (derived from our own work).

The mean age and BMI of the 10520 participants in the monastery cohort study were 51.52 ± 8.90 years and 28.17 ± 5.76 kg/m^2^, respectively. Of the participants, 5,633 (53.6%) were female, 5907 (56.1%) were rural, 9527 (90.6%) were married, 1738 (16.5%) were illiterate, and 3435 (32.7%) were obese (demographic characteristics are presented in Table 1). In this study, the prevalence of metabolic syndrome (95% CI) according to IDF and ATP definition was 42.87% (41.92–43.81) and 40.68% (39.74–41.62), respectively (Table 2). The geographic distribution of metabolic syndrome based on the IDF definition in Some‚e Sara is presented in Figure 1.

According to the descriptions mentioned in this method, all results and analyzes were performed according to IDF definition in this study. The prevalence of the syndrome components in the population was 75.8% for central obesity, 43.1% higher triglyceride, 40.6% lower HDL cholesterol, 39.2% fasting blood glucose, and 37.9% high blood pressure. The prevalence of metabolic syndrome was 2.87% and 62.03% in low weight and obese subjects, respectively.

Investigating the relationship between metabolic syndrome and demographic characteristics in univariate regression analysis showed that, with increasing age, the prevalence of the syndrome increased. The prevalence of the syndrome in the age group 45–54 was 1.4 folds more than the age group 45 (OR = 1.40, 95% CI: 1.26—1.55, *P* ≤ 0.001), which is 2.13 folds (OR = 2.13, 95% CI: 1.84—2.47, *P* ≤ 0.001) in the age group of 65–70 years. Multivariate analysis also showed an increase in the prevalence of metabolic syndrome with age and all of these relationships were statistically significant (*P* < 0.001). The prevalence of metabolic syndrome in women was 3.2 times more than males in univariate and multivariate regression analysis (OR = 3.23, 95% CI: 2.98—3.51, *P* ≤ 0.001).

The prevalence of metabolic syndrome was 18% lower on average in the rural population (OR = 0.82, 95% CI: 0.76—0.89, *P* ≤ 0.001). In multivariate analysis, rural residents also had the least prevalence (*P* < 0.001).In terms of marital status, married people had a 33% lower prevalence of the syndrome than single individuals, which was not significant after adjusting for other variables. In terms of education, the prevalence of the syndrome decreases with the increase of education. The prevalence of the syndrome was 31% lower in the elementary education than in the illiterate population, which was less than 50% in the academic population (*P* < 0.001), but after adjusting for other variables, although the prevalence of the syndrome continued to decrease with education, the relationships did not reach a significant level. The prevalence of metabolic syndrome was also 59% lower in those currently employed (<0.001), but after adjusting for other variables, although prevalence of the syndrome was 8% lower in employees, it did not reach a significant level (Table 3). Demographic variables such as age, gender, and place of residence were identified as strong and independent predictors of metabolic syndrome in both single and multivariate regression analysis.

The prevalence of the components of the syndrome according to demographic characteristics showed that the prevalence of central obesity was almost the same in all age groups. And, over three quarters of people in every age group have central obesity. Hyperglycemia and hypertension have also increased with age. The decreasing trend of HDL cholesterol has also been shown to decrease with age. Also, while half of the men had central obesity, it was more than 98% in females (prevalence of components in terms of demographic characteristics is shown in Table 4).

**Figure 2:**
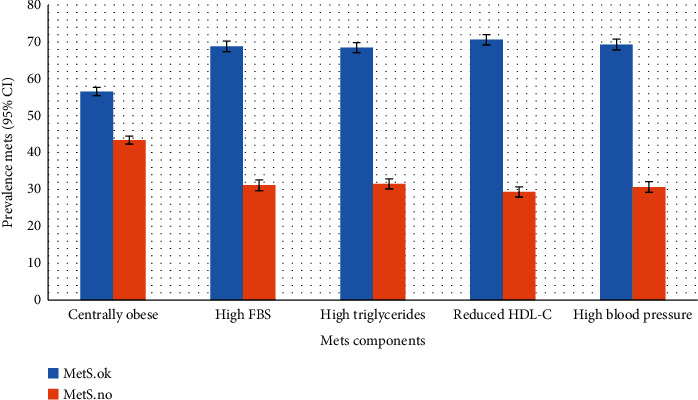
The prevalence of each components of metabolic syndrome in our study.

Because a person has a component of the syndrome, at the same time, a few percent of the study population has a metabolic syndrome. The results showed that the prevalence of metabolic syndrome in those with central obesity was 56.59%, while the prevalence of obesity in those without syndrome was 43.40%. Also, individuals with high blood sugar, high triglyceride, lower HDL cholesterol, and high blood pressure had metabolic syndrome (68.82%, 68.45%, 70.64%, and 69.31%, respectively) (Figure 2).

More than 92% of the population had at least one component of the syndrome. This index was 84.5% in men and more than 99% in women. Also, 3% of men and 7.4% of women had all five components (5.4%). In all cases, the most prevalent cases were those with only two (27.1%) and three components (24.8%), respectively.

## 4. Discussion

This study showed the prevalence of the syndrome and its components based on the most important demographic characteristics. The prevalence of the syndrome according to IDF was 42.87%. Demographic variables such as age, gender, and place of residence were identified as strong and independent predictors of metabolic syndrome. Among the syndrome components, central obesity had the highest prevalence (75.8%). Over 92% of the population also had at least one component of the syndrome. This prevalence was 34.1% among adults over 20 years in the United States [30], in Taiwan 36.4% [31], and in the Cuschieri study in a population of 18 to 70 years old in Malta, it was 26.30% [32]. Mokhayeri et al., using a systematic review and meta-analysis reported the prevalence of this syndrome 28% for both IDF and ATP index [33]. Delavari et al. in a national study of 30 Iranian provinces reported this rate 34.7% and 37.4% by ATP and IDF criteria [34]. According to the study of Hajian-Tilki et al. in northern Iran, the prevalence of metabolic syndrome was 42.3% (36.5% in males and 47.3% in females) according to ATP III definition [21]. The rate of metabolic syndrome in our study was higher than other studies and was in line with similar study in northern Iran. The prevalence of the syndrome in northern Iran appears to be higher than elsewhere, and further research is needed on the specific lifestyle of people in these areas. These differences in prevalence can also be attributed to lifestyle, genetic factors, age, and sex structures of the population under study. It can also vary depending on the definition used to diagnose it [35].

Obesity is one of the main factors among the components of the metabolic syndrome. In this study, central obesity had the highest prevalence (75.8%), and this index was more than 98% in females. The prevalence of this syndrome in adults continues to increase as the prevalence of obesity has increased dramatically [36]. Obesity is emerging as a leading cause of premature death in the United States and around the world [37]. In the Noshad et al.'s study, the prevalence of obesity in the Iranian population was 52.48% [38], and in the Ortiz-Rodrguez's study, 65.4% had central obesity. Currently, more than half of Iran's adult population has central obesity, which emphasizes the need for comprehensive preventive strategies to reduce energy consumption [39].

With increasing age, the prevalence of the syndrome increases, reaching more than double in the elderly compared to the age group below 45 years, which is a strong independent predictor of metabolic syndrome. According to the National Health and Nutrition Survey (NHNS) in American adults, the prevalence of the syndrome has increased with age from 20–29 years to 60–69 years [40]. In other studies, the prevalence of the syndrome has increased with age [32, 41, 42]. This increase in age may be due to decreased physical activity and increased obesity in older people. Therefore, older people should be prioritized for disease screening and health education.

The prevalence of the syndrome in women was three times higher than in men, and this variable was a strong and independent predictor of metabolic syndrome. In general, the prevalence of the syndrome in women is higher than in men [43], and in some studies, this amount was more than double in women [44] which is in line with the results of this study. However, in some studies, the prevalence of metabolic syndrome was higher in men than in women [45, 46]. This increase may also be due to low mobility in Iranian women.

In terms of residence, the prevalence of the syndrome was higher in urban areas, and this variable was a strong and independent predictor of metabolic syndrome. According to the results of the study by Cozma et al., the rural environment was a risk factor for metabolic syndrome [47]. In Noshad's study, the prevalence of this syndrome and its five components was significant for everyone in terms of location and was higher in cities for all components [38] which is consistent with the results of our study. In northern Iran and especially in Guilan province, this may be justified due to physical activity caused by agricultural activity, especially in rural areas. But these changes appear to be less pronounced in the coming years with diet changes, not only in urban areas but also in rural communities due to the modernization of Iranian society [48].

The prevalence of the syndrome decreases with increasing education. In the Ebrahimi study, the regression model showed that the prevalence of the syndrome decreased significantly as education increased [42]. In other studies, people with high school and higher education were less likely to have the syndrome component than those with secondary and elementary education [49], but there was no significant relationship between education and the syndrome [50]. As education increases, people are increasingly adopting health behaviors such as not smoking and engaging in vigorous physical activity [51, 52]. It is also possible that people with higher education will have a healthier lifestyle because of their higher health literacy.

More than 92% of the population also had at least one component of the syndrome. In a study in the Iranian capital, 88% of adults had at least one of the components of the metabolic syndrome [53].

### 4.1. Strengths and Limitations

The high sample size and population-based design were the main strengths of this study. Because we have complete information about disease records and treatment to define the syndrome, having current disease and medications such as antihypertensive or anti-diabetic drugs are included in the definition, which would be a more realistic estimate. One of the most important limitations of this study is the design of the cross-sectional study as well as lack of information about the changes in metabolic syndrome and its components.

## 5. Conclusion

Following prolonged health reforms in Iran, the prevalence of communicable diseases is decreasing and the prevalence of noncommunicable diseases and its risk factors is increasing. Preliminary results indicate a high prevalence of metabolic risk factors for cardiovascular disease in Guilan province. The prevalence of risk factors was higher among men, older people, illiterate, single, and unemployed participants. Complex relationships between the components of the syndrome in subsequent phases of Persian cohort are necessary because longitudinal studies are needed to evaluate causal relationships. It is essential to educate healthy lifestyle behaviors and further health education in high-risk groups identified in this study.

## Figures and Tables

**Table 1:** Characteristics of demographic variables and prevalence of metabolic syndrome according on IDF definition (*n* = 10520).

Variables	Category	*N* (%)	Prevalence MetS (95% CI)
Age (years)	44–35	3142 (29.9)	33.63 (31.89–35.41)
	45–54	3852 (36.6)	41.53 (39.99–43.09)
	55–65	2730 (25.9)	50.33 (48.50–52.15)
	65–70	796 (7.6)	51.97 (48.89–55.04)

Gender	Men	4887 (46.4)	28.01 (26.77–29.29)
	Women	5633 (53.6)	55.75 (54.44–57.04)

Location	Urban	4613 (43.9)	45.50 (44.06–46.94)
	Rural	5907 (56.1)	40.82 (39.57–42.07)

Marital status	Single	993 (9.4)	51.66 (48.54–54.76)
	Married	9527 (90.6)	41.95 (40.96–42.94)

Education Level	Illiterate	1738 (16.5)	52.42 (50.06–54.77)
	1–5 years	3312 (31.5)	43.45 (41.77–45.15)
	6–12 years	4832 (45.9)	39.90 (38.52–41.29)
	Academic level	638 (6.1)	35.70 (32.05–39.51)

Current employment	No	4781 (45.4)	54.61 (53.19–56.01)
	Yes	5739 (54.6)	33.08 (31.88–34.31)

BMI	Low weight	141 (1.4)	2.87 (1.08–7.44)
	Normal	2746 (26.0)	15.47 (14.16–16.88)
	Overweight	4198 (39.9)	46.44 (44.93–47.95)
	Obesity	3435 (32.7)	62.03 (60.39–63.63)

**Table 2:** Definitions and frequency of metabolic syndrome and its components.

Components		Definitions	Scale	Prevalence
Metabolic syndrome		International Diabetes Federation (IDF)	Having the obesity component in addition to two of the other four components	4510 (42.9)
		Adult Treatment Panel (ATP)III	Three of the five components of metabolic syndrome	4280 (40.7)

Components of metabolic syndrome	Central obesity	IDF	Men ≥94 cm Women ≥80 cm	7969 (75.8)
		ATP	Men ≥102 cm Women ≥88 cm	6250 (59.4)
	Blood glucose	All definitions	≥100 mg/dl or previous diagnosis of type 2 diabetes	4119 (39.2)
	High triglyceride	All definitions	≥150 mg/dl or a dedicated treatment for these fat disorders	4537 (43.1)
	HDL cholesterol	All definitions	<40 mg/dl men; <50 mg/dl women or a dedicated treatment for these fat disorders	4269 (40.6)
	Hypertension	All definitions	Systolic BP ≥130 mmHg; diastolic BP ≥85 mmHg or the treatment of previous blood pressure detected	3983 (37.9)

**Table 3:** The relationship between metabolic syndrome and demographic characteristics in single and multivariate regression analyses.

Variables	Category	OR (crude)		OR (adjusted)	
		OR (95% CI)	*P* value	OR (95% CI)	*P* value
Age (years)	44–35	Ref			
	45–54	1.40 (1.26–1.55)	<0.001	1.42 (1.28–1.59)	<0.001
	55–65	1.99 (1.79–2.22)	<0.001	2.10 (1.86–2.36)	<0.001
	65–70	2.13 (1.84–2.47)	<0.001	2.32 (1.96–2.74)	<0.001

Gender	Men	Ref			
	Women	3.23 (2.98–3.51)	<0.001	3.23 (2.89–3.61)	<0.001

Location	Urban	Ref			
	Rural	0.82 (0.76–0.89)	<0.001	0.73 (0.67–0.80)	<0.001

Marital status	Single	Ref			
	Married	0.67 (0.59–0.77)	<0.001	1.06 (0.92–1.22)	0.387

*Education Level*	Illiterate	Ref			
	1–5 years	0.69 (0.62–0.78)	<0.001	0.90 (0.79–1.02)	0.120
	6–12 years	0.60 (0.53–0.67)	<0.001	0.88 (0.77–1.00)	0.055
	Academic level	0.50 (0.41–0.60)	<0.001	0.82 (0.66–1.01)	0.064

*Current employment*	No	Ref			
	Yes	0.41 (0.38–0.44)	<0.001	0.92 (0.83–1.03)	0.179

**Table 4:** Prevalence of metabolic syndrome components by demographic characteristics based on IDF definition.

Variables	Category	Central obesity	FBS	High triglyceride	HDL cholesterol	Hypertension
Age (years)	44–35	75.41 (73.77–76.98)	26.43 (24.81–28.10)	41.67 (39.84–43.52)	43.30 (41.46–45.15)	18.21 (16.81–19.69)
	45–54	76.55 (75.19–77.86)	35.97 (34.47–37.50)	44.11 (42.56–45.69)	41.27 (39.73–42.83)	33.18 (31.71–34.68)
	55–65	75.11 (73.49–76.65)	50.74 (48.91–52.57)	44.42 (42.61–46.24)	38.82 (37.05–40.62)	53.73 (51.90–55.55)
	65–70	75.39 (72.64–77.95)	53.06 (49.97–56.12)	39.62 (36.65–42.67)	35.47 (32.58–38.47)	64.22 (61.22–67.12)
	*P* value	0.523	<0.001	0.012	<0.001	<0.001

Gender	Men	50.00 (48.59–51.40)	36.14 (34.80–37.50)	45.64 (44.24–47.04)	26.46 (25.24–27.71)	35.03 (33.71–36.38)
	Women	98.08 (97.69–98.41)	41.76 (40.48–43.05)	40.94 (39.66–42.23)	52.82 (51.51–54.12)	40.30 (39.03–41.59)
	*P* value	<0.001	<0.001	<0.001	<0.001	<0.001

Location	Urban	77.47 (76.24–78.66)	38.46 (37.07–39.88)	46.35 (44.91–47.79)	48.13 (46.69–49.57)	36.81 (35.43–38.21)
	Rural	74.40 (73.28–75.50)	39.68 (38.44–40.49)	40.61 (39.37–41.87)	34.70 (33.49–35.92)	38.67 (37.44–39.92)
	*P* value	<0.001	0.203	<0.001	<0.001	0.051

Marital status	Single	87.10 (84.87–89.05)	43.50 (40.44–46.61)	39.37 (36.37–42.45)	49.24 (46.14–52.35)	42.69 (39.65–45.80)
	Married	74.56 (73.68–75.43)	38.70 (37.72–39.68)	43.51 (42.52–44.51)	39.67 (38.69–40.66)	37.35 (36.39–38.33)
	*P* value	<0.001	0.003	0.012	<0.001	0.001

Education level	Illiterate	80.42 (78.49–82.22)	51.55 (49.20–53.90)	41.10 (38.81–43.44)	39.08 (36.81–41.40)	52.36 (50.01–54.71)
	1–5	77.43 (75.98–78.83)	39.91 (38.25–41.59)	42.88 (41.20–44.57)	40.64 (38.97–42.32)	37.25 (35.61–38.91)
	6–12	74.14 (72.89–75.36)	34.87 (33.53–36.22)	43.39 (41.99–44.79)	40.98 (39.59–42.37)	33.14 (31.82–34.48)
	Academic	66.35 (62.57–69.93)	33.01 (29.45–36.78)	47.70 (43.83–51.60)	40.60 (36.83–44.47)	36.33 (32.67–40.16)
	*P* value	<0.001	<0.001	0.037	0.589	<0.001

Current employment	No	93.05 (92.29–93.74)	44.44 (43.04–45.85)	41.72 (40.33–43.13)	49.98 (48.57–51.40)	43.54 (42.14–44.95)
	Yes	61.33 (60.06–62.58)	34.74 (33.52–35.98)	44.29 (43.01–45.58)	32.74 (31.53–33.96)	33.12 (31.91–34.35)
	*P* value	<0.001	<0.001	0.008	<0.001	<0.001

Total		75.75 (74.92–76.56)	39.15 (38.22–40.09)	43.12 (42.18–44.07)	40.57 (39.64–41.52)	37.86 (36.93–38.79)

## Data Availability

The datasets analyzed during the current study are available from the corresponding author on reasonable request.
